# Homing to solid cancers: a vascular checkpoint in adoptive cell therapy using CAR T-cells

**DOI:** 10.1042/BST20150254

**Published:** 2016-04-11

**Authors:** Ann Ager, H. Angharad Watson, Sophie C. Wehenkel, Rebar N. Mohammed

**Affiliations:** * Systems Immunity University Research Institute and Division of Infection and Immunity, School of Medicine, Cardiff University, Cardiff, CF14 4XN, U.K.

**Keywords:** chemokine receptors, endothelial cell anergy, extravasation, high endothelial venules, homing, integrins, selectins, tumour blood vessels, tumouricidal T-lymphocytes, vessel normalization

## Abstract

The success of adoptive T-cell therapies for the treatment of cancer patients depends on transferred T-lymphocytes finding and infiltrating cancerous tissues. For intravenously transferred T-cells, this means leaving the bloodstream (extravasation) from tumour blood vessels. In inflamed tissues, a key event in extravasation is the capture, rolling and arrest of T-cells inside blood vessels which precedes transmigration across the vessel wall and entry into tissues. This depends on co-ordinated signalling of selectins, integrins and chemokine receptors on T-cells by their respective ligands which are up-regulated on inflamed blood vessels. Clinical data and experimental studies in mice suggest that tumour blood vessels are anergic to inflammatory stimuli and the recruitment of cytotoxic CD8^+^ T-lymphocytes is not very efficient. Interestingly, and somewhat counter-intuitively, anti-angiogenic therapy can promote CD8^+^ T-cell infiltration of tumours and increase the efficacy of adoptive CD8^+^ T-cell therapy. Rather than inhibit tumour angiogenesis, anti-angiogenic therapy ‘normalizes’ (matures) tumour blood vessels by promoting pericyte recruitment, increasing tumour blood vessel perfusion and sensitizing tumour blood vessels to inflammatory stimuli. A number of different approaches are currently being explored to increase recruitment by manipulating the expression of homing-associated molecules on T-cells and tumour blood vessels. Future studies should address whether these approaches improve the efficacy of adoptive T-cell therapies for solid, vascularized cancers in patients.

## Introduction

Retrospective studies of resected primary tumours from colorectal cancer patients were the first to show that the presence and location of tumour-infiltrating T-lymphocytes are prognostic for patient outcome [1]. Specifically, high densities of cytotoxic and memory T-cells within the tumour and at the invasive tumour margin correlate with longer disease-free survival and overall patient survival. These findings have been extended to many cancers including melanoma, breast, head and neck, gastric and lung [2]. How cytotoxic and memory T-cells home to and infiltrate cancerous tissues are not understood. Blood vessels which are especially adapted to recruit lymphocytes from the bloodstream into lymph nodes, so-called high endothelial venules (HEV), have been reported in solid cancers [3,4]. Further, the presence of HEV blood vessels in resected, primary breast cancer and melanoma correlate with improved patient outcome. Although it is not known if tumour induced HEV are the entry portal for cytotoxic and memory T-cells, these findings highlight the critical role that tumour blood vessels play in orchestrating an effective immune response by recruiting T-cells from the bloodstream into cancerous tissues. A major goal of cancer immunotherapy is to generate tumouricidal T-cells that are able to overcome tumour-induced immunosuppression and kill cancer cells. An equally important goal of cancer immunotherapy will be to match homing addresses on tumouricidal T-cells with vascular addresses on tumour blood vessels in order to recruit blood-borne T-cells into cancerous tissues.

## The advent of adoptive T-cell therapies to treat cancer patients

Tumour-specific T-cells isolated from cancer patients recognize and kill tumours *in vitro* yet the same cells fail to eradicate cancer in the patient due to tumour-induced immunosuppression. Early attempts to overcome immunosuppression included isolating tumour infiltrating lymphocytes (TILs) from resected melanoma lesions, expanding tumour-reactive T-cells and infusing large numbers back to patients with progressive metastatic melanoma [5]. These ground-breaking clinical studies have resulted in objective tumour regression in >50% of patients and were the first to demonstrate that adoptive cell therapy (ACT) using tumouricidal T-lymphocytes could be used to treat cancer patients. Autologous T-cells used for ACT have been extended to peripheral blood T-cells genetically modified to express MHC-restricted, high affinity tumour-specific TCR (TCR_gm_) to overcome dominant immunosuppression in the cancer patient [6]. The recent remarkable clinical progress using re-directed T-cells expressing a non-MHC restricted chimaeric antibody receptor (CAR) that binds to CD19 on B-cells for the treatment of patients with, otherwise refractory, B-cell malignancies has highlighted the potential of CAR T-cells to treat a wide range of solid cancers [7–9]. However, there are inherent and perceived difficulties in using CAR T-cells to target solid cancers, particularly the identification of target antigens that are selectively expressed by cancers and not normal tissues. The ability of CAR T-cells to overcome counter-attack by the tumour as well as local immununosuppression are also important (see Watson et al. SHP-1; the next checkpoint target for cancer immunotherapy? in this issue). Of equal importance is the ability of CAR T-cells to home to and infiltrate cancerous tissues which is the subject of this review. Objective tumour regression of metastatic melanoma using autologous T-cells implies that transferred T-cells homed to the cancer, but this therapy does not work in all patients. It will be important to determine how T-cell homing to solid cancers is linked to the outcome of ACT if this type of immunotherapy is to move beyond patient-based early clinical trials and into clinical practice.

## Designer adoptive T-cell therapy for solid cancers

**Figure 1:**
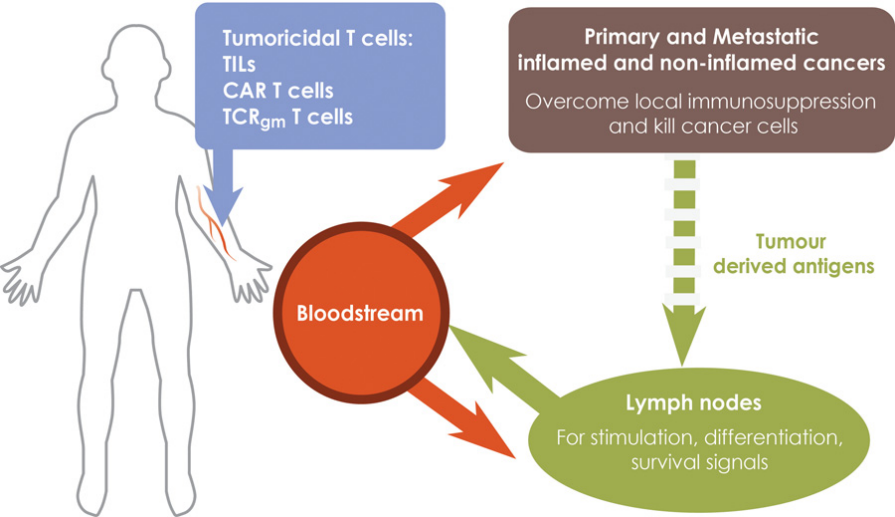
A designer adoptive T-cell therapy for solid cancers T-lymphocytes expressing conventional TCRs (TILs, TCR_gm_) or CARs at different stages of activation and differentiation are required to kill primary and metastatic cancers and to persist in cancer patients. Fully activated, tumouricidal T-cells expressing inflammation-associated homing molecules migrate from tumour blood vessels into primary cancers and sites of metastases (including sentinel lymph nodes) where they kill cancer cells. Tumouricidal T-cells migrating to non-cancerous tissues are unable to exert anti-cancer activity and are ineffective. Central memory T-cells expressing conventional TCRs, but not CARs, are re-activated by endogenously processed and presented tumour-derived antigens in tumour-draining lymph nodes before being redistributed to cancerous tissue. Central memory T-cells receive survival signals during normal recirculation through lymphoid organs. Recruitment of T-cells into non-inflamed cancers is promoted by patient conditioning which sensitizes the normally anergic tumour blood vessels to inflammatory mediators, increases the expression of homing-associated molecules and promotes recruitment of tumouricidal T-cells. Maturation of tumour blood vessels by activated T-cells in already inflamed tumours, promotes the development of HEV which recruit central memory T-cells into the tumour and shift the site of T-cell activation to cancerous tissues which avoids the loss of tumouricidal T-cells to non-cancerous tissues during their redistribution from the normal LN site of priming. γ-chain cytokines generate central memory and effector T-cells during T-cell expansion prior to adoptive transfer to cancer patients.

An ideal adoptive T-cell therapy is that tumouricidal T-cells (CAR, TIL or TCR_gm)_ injected into the bloodstream are recruited into cancerous tissues to bring about cancer cell killing (Figure 1). An additional requirement is that transferred T-cells home to lymph nodes where survival signals promote long-term persistence. Homing to sentinel lymph nodes is necessary to kill lymph node metastases and may be critical to re-stimulate effector function in TIL and TCR_gm_ T-cells by endogenously processed and presented tumour-derived antigens, but not for CAR T-cells which bind to native cell surface antigens. One way of achieving dual homing to cancerous tissues and lymph nodes is exploit the fact that T-cells at different stages of activation home to different types of tissues.

**Figure 2:**
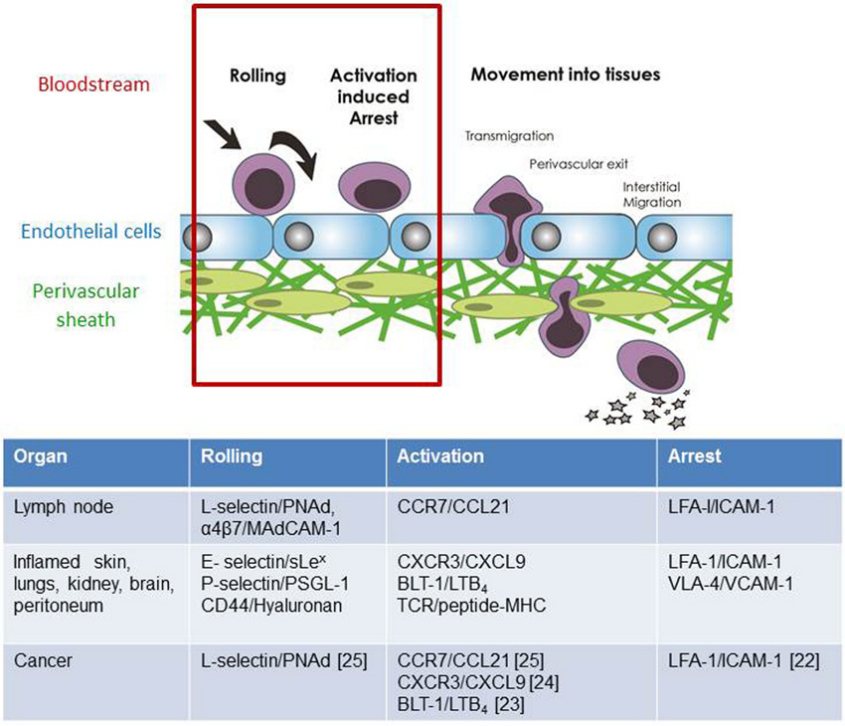
Homing addresses for T-cell entry into normal and cancerous tissues T-lymphocyte recruitment from the bloodstream into tissues is regulated by a co-ordinated sequence of adhesive interactions which can be separated into distinct stages. The term ‘homing address’ (red box) describes the combination of rolling, activation and arrest receptors on T-cells and their respective ligands on the apical (inner) surface of blood vessel EC. Distinct homing addresses for T-cells according to the stage of T-cell activation, nature of the inflammatory stimulus and the organ involved have been identified and are listed. For example, the homing address on naïve and central memory T-cells for lymph nodes is L-selectin-CCR7-LFA-1 integrin. Specifically, L-selectin (CD62L) on T-cells binds to PNAd on the apical, inner surface of HEV blood vessels and allows T-cells to roll on the inner blood surface in the direction of blood flow. Rolling T-cells encounter immobilized CCL21 on the EC surface which engages CCR7 on T-cells and arrests rolling T-lymphocytes by activating LFA-1 integrin binding to ICAM-1. Activated Th_1_ CD4 T-cells entering inflamed tissues such as hypersensitivity lesions in the skin use a different homing address. Here the rolling receptor is E-selectin which is up-regulated on vascular EC by inflammatory cytokines. Inflammatory chemokines synthesized by inflamed tissues are reverse transcytosed across the blood vessel wall and immobilized on the apical surface of EC where they arrest rolling T-cells via LFA-1 and/or VLA4 integrins. Tumour blood vessels express homing-associated adhesion molecules and chemokines typical of inflamed tissues and lymph nodes and those which have been shown to regulate the recruitment of adoptively transferred tumouricidal T-cells from tumour blood vessels into tumours in syngeneic mice are cited.

Naive and central memory T-lymphocytes continuously recirculate through lymph nodes via HEV and lymphatics where they screen dendritic cells for antigenic peptides [10,11]. Following engagement of TCR and the induction of proliferation, activated lymphocytes re-gain access to the bloodstream and migrate to non-lymphoid tissues, particularly sites of inflammation (Figure 2). However, activated CD4^+^ T-cells also localize to non-lymphoid tissues such as the lungs, liver, gut and salivary glands in the absence of ongoing inflammation [12,13]. Following primary infection with virus or bacteria, activated CD8^+^ T-cells are found at sites of virus replication where effector T-cells differentiate into tissue resident memory cells and play a key role in protection against re-infection [14]. Activated CD8^+^ T-cells also migrate to non-lymphoid organs such as the liver, lungs, lamina propria, fatpad, peritoneal cavity and in some cases, the CNS even though they were not the primary sites of virus replication in these studies [15,16]. If the homing properties of pathogen-specific effector T-cells can be reproduced in CAR or TCR_gm_ T-cells, their widespread dissemination following adoptive transfer into the bloodstream would be an advantage to target primary as well as metastatic disease. Lymph node homing properties to promote persistence of transferred T-cells will depend on limiting T-cell differentiation during *in vitro* expansion to maintain a central memory phenotype [17]. Naive and self-renewing T-cells generated from autologous haemopoietic or induced pluripotential stem cells provide additional sources of T-cells for adoptive T-cell therapy which offer superior efficacy due to their ability to recirculate through lymphoid organs.

## Homing addresses for T-lymphocyte recruitment from the bloodstream into tissues

Recruitment from the bloodstream into tissues involves a sequence of adhesive interactions between T-lymphocytes and cells of the blood vessel wall which can be divided into distinct stages of rolling, activation-induced arrest and movement into tissues (Figure 2). It is a rapid process taking up to 10 min to complete and the ability to image the behaviour of T-cells during rolling and activation-induced arrest inside small blood vessels has allowed the adhesion molecules and signalling events that regulate these stages to be identified. The subsequent movement of T-cells into the tissue parenchyma is regulated independently of activated-induced arrest inside blood vessels. For example, T-lymphocytes accumulate in the walls and perivascular sheath of blood vessels (so-called perivascular cuffing) when entry into the tissue parenchyma is prevented [18,19].

A key event in T-cell homing is the selection of T-lymphocytes from the total pool of circulating leucocytes on to the vessel wall under the shear stresses generated by flowing blood. This occurs primarily in post-capillary venules where haemodynamic forces promote margination of leucocytes to the outside stream of flowing blood. The term ‘homing address’ describes the combination of rolling, activation and arrest receptors on T-cells and their respective ligands or ‘vascular address’ expressed on the apical (inner) surface of blood vessel endothelial cells (EC) that directs entry into tissues. Homing addresses for lymph nodes and sites of inflammation in the skin and other organs have been identified (Figure 2). The considerable heterogeneity in vascular endothelium between organs, at different locations in the vascular bed and in T-cell subsets means that there are distinct homing addresses for T-cells according to the stage of T-cell activation, nature of the inflammatory stimulus and the organ involved [10]. Although the general rule is that homing addresses on T-cells comprise a selectin–chemokine receptor–integrin combination, there are exceptions (Figure 2). One important exception is that TCR recognition of peptide–MHC complexes on the inside walls of blood vessels can substitute for chemokine receptors and induce integrin-dependent arrest of rolling T-cells [20]. Antigen can be presented either by perivascular dendritic cells, which extend protrusions into the vessel lumen [20], or by EC [21]. Following the recruitment of T-cells expressing cognate TCR, the release of inflammatory cytokines activates local blood vessels which recruit non-cognate TCR (so called 3rd party or bystander T-cells) in a chemokine-dependent manner using a conventional selectin–chemokine receptor–integrin homing address [20].

Some of the adhesion molecules and chemokines that regulate T-lymphocyte homing into sites of inflammation and/or lymph nodes have been implicated in homing to cancers in experimental mice [22–25]. Knowledge of selectin, integrin and chemokine receptors expressed by infiltrating cytotoxic T-lymphocytes and their respective ligands in cancerous and normal tissues will assist in the selection of appropriate cancer homing addresses for expression in CAR or TCR_gm_ T-cells. For example, transduction of CAR T-cells with chemokine receptors for human tumour cell-derived chemokines increases homing to solid cancers and controls their growth in immunodeficient mice [26,27].

## Lessons about T-lymphocyte homing from experimental animal models and early phase clinical studies of adoptive T-cell therapies to treat solid cancers

**Figure 3:**
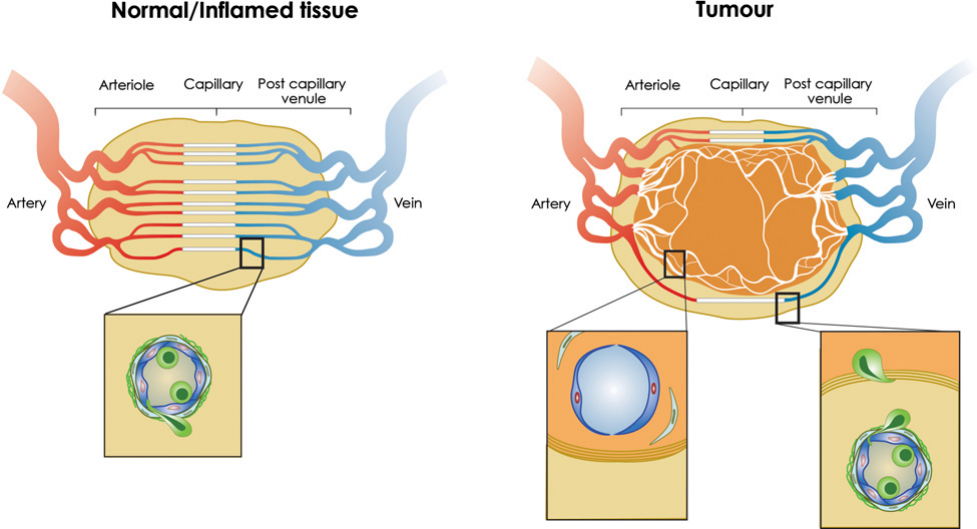
T-cell recruitment from normal, inflamed and tumour blood vessels (**Left**) In normal or inflamed tissues, the vascular network is highly ordered and T-cells are recruited from post-capillary venules where haemodynamic forces promote the margination of T-cells and other leucocytes to the outside stream of flowing blood. T-cell recruitment from HEV in lymph nodes or inflamed blood vessels in non-lymphoid tissues follows the multistep adhesion cascade described in Figure 2. Inset shows T-cell (green) binding to and in the process of transmigrating the EC lining and surrounding pericytic sheath which together comprise the blood vessel wall. (**Right**) Angiogenic blood vessels growing inside the tumour in response to hypoxia lack pericyte support and are highly disorganized, poorly perfused and leaky. They are non-responsive (anergic) to inflammatory stimuli but responsive to tumour-derived factors that prevent expression of homing molecules on tumour EC and induce FasL expression which directly kills tumouricidal T-cells. As a consequence intra-tumoural blood vessels are poor at recruiting effector CD8^+^ T-cells into the tumour [30]. In contrast, peritumoural blood vessels respond to inflammatory stimuli, up-regulate homing-associated molecules and recruit effector CD8^+^ T-cells to the peritumoural area. Cognate recognition of tumour antigen by conventional TCR regulates the movement of extravasated T-cells into the tumour to kill cancer cells. Insets show (left) immature, anergic tumour blood vessel lacking pericyte support inside the tumour and (right) mature peritumoural vessel supporting T-cell recruitment adjacent to a fibroblast-associated matrix which T-cells cross to enter the tumour. Chemokine production by cancer-associated fibroblasts can limit tumouricidal T-cell entry and killing of tumour tissue by arresting T-cells in the peritumoural area [30].

Early studies of leucocyte (neutrophil) behaviour inside blood vessels of subcutaneous tumours in immunodeficient mice demonstrated lower levels of rolling and arrest than in normal skin, even after administration of tumour necrosis factor-α (TNF-α) or LPS, which suggested that tumour blood vessels are anergic to inflammatory stimuli [28,29]. Subsequent studies demonstrated clearly that effector CD8^+^ T-lymphocytes do not spontaneously roll or undergo activation induced arrest in tumour blood vessels even following LPS administration [22]. The fact that tumour blood vessels are poor at recruiting CTLs has suggested that they represent a vascular checkpoint which limits the success of ACT, as well as other immunotherapies such as vaccination and checkpoint inhibition [22,30]. A number of different mechanisms underlie poor CTL recruitment, not least of which is that tumour blood vessels are highly disorganized, immature, lacking an organized pericytic sheath and poorly perfused (Figure 3). Low CD8^+^ T-cell flux through tumour blood vessels, absence of shear stresses necessary for recruitment and/or inefficient chemokine presentation could all contribute to poor CTL recruitment. Additional mechanisms include tumour-derived products which restrict expression of homing molecules, such as ICAM-1, on tumour EC [31] and induce expression of FasL [32]. EC anergy may be related to the pro-angiogenic tumour environment since angiogenic growth factors prevent cytokine induced homing molecule expression by EC [33].

Adoptive T-cell therapy does restrict the growth of solid vascularized cancers in experimental mice and in patients so, if tumour blood vessels are poor at recruiting injected T-cells, how does it work? Intravital imaging has demonstrated that tumour specific T-cells are recruited, not from blood vessels within the tumour nest but, from blood vessels in the peritumoural area where homing-associated adhesion molecules are preferentially expressed [34] (Figure 3). Recruitment from blood vessels is not dependent on TCR recognition of cognate tumour antigen and, most likely, reflects the local inflammatory environment which is not restrained by the adjacent tumour environment. However, the motility of CTLs and their ability to infiltrate the tumour mass does depend on cognate recognition of tumour antigen by TCR [35,36]. The density of infiltrating cytotoxic and memory T-cells at the invasive tumour margin as well as within the tumour of cancer patients correlate with overall patient survival [2]. Preclinical studies showing that T-cells exit peritumoural blood vessels before invading tumour tissue support the clinical observations. However, whether T-cells are recruited directly into the tumour nest or indirectly from the peritumoural area may depend on the location and size of the tumour. The efficacy of CAR or TCR_gm_ T-cell therapy for solid tumours is cell dose dependent [37,38] so understanding what controls the number of tumouricidal T-cells that accumulates inside tumour tissues is important because this correlates with patient survival. If MHC-restricted TCR signalling is required for T-cell infiltration of cancerous tissues, it will be important to determine whether signalling pathways downstream of CARs substitute for TCR signalling or whether there is an obligatory role for the canonical TCR in this process. The fact that CAR expressing human T-cells migrate to and control the growth of human tumours in immunodeficient mice does not provide an answer to the important question of whether CARs can fully substitute for a conventional TCR in T-cell homing since xenoreactivity as well as alloreactivity (if T-cell and tumours are not MHC-matched) could regulate T-cell infiltration of tumours in these mouse models. The preclinical studies on T-cell homing to cancerous tissues described above have used transplanted antigen-expressing tumours and high numbers of transgenic T-cells specific for transfected antigens. Therefore, analysis of spontaneous mouse tumours relevant to human cancers using CARs and TCR_gm_ T-cells recognizing relevant tumour antigens will be required before applying the rules governing tumouricidal T-cell homing in transplanted syngeneic or xenogeneic tumours to human cancers.

There have been very few clinical studies of T-cells following injection into the venous circulation of cancer patients. However, an early study showed that ^111^indium-labelled TILs homed to melanoma lesions and TIL homing was associated with objective clinical responses in 38% (10/26) patients [39]. In a phase I study of CAR T-cells in ovarian cancer, accumulation of CAR T-cells in a pelvic mass (presumed cancer) was seen in a single patient out of the seven treated but there were no clinical responses in any patient [40]. These results raise the possibility that the lack of clinical response is related to lack of T-cell homing to the cancer. Studies in mice have shown that HEV expression of the arrest chemokine, CCL21, is down-regulated in tumour-draining (sentinel) lymph nodes and T-lymphocyte recruitment is consequently reduced [41], which, in cancer patients, will affect T-cell re-stimulation and survival. Whole body imaging showed that CAR T-cells localized in the lungs and then the liver and spleen in 7/7 patients. Interestingly, lung localization persisted for longer when re-stimulated T-cells were transferred [40]. In experimental mice, tumour-specific CD8^+^ T-cells traffic indiscriminately to lymph nodes, liver, spleen and lungs in tumour-bearing mice while still being able to control tumour growth [42]. This finding suggests that the ability of transferred T-cells to home to cancerous tissues may not be limited by widespread distribution to non-cancerous tissues, which has been the conclusion thus far.

Indiscriminate homing of CAR T-cells, TILs and TCR_gm_ may, however, contribute to their lack of persistence which has been repeatedly reported in patients and experimental mouse models. This may be due to lack of encounter with cognate antigen following homing to non-cancerous tissues and/or lack of homing to lymph nodes where survival and/or reactivation signals are presented. Inclusion of CD28 or other co-stimulator signalling domains in so-called second and third generation CARs prolongs the persistence of CAR T-cells but whether this is related to lymph node homing remains to be determined. Checkpoint blockade inhibitors and host-conditioning using fludarabine and cyclophosphamide increase the efficacy of TILs, CAR and TCR_gm_ T-cells but whether this is related to altered homing is also not known [8,43–45]. However, strategies to boost the efficacy of CAR T-cells will need to be weighed against the risks of clonal deletion or lymphoma development and increased incidence of unrelated cancers [46].

## Overcoming vascular checkpoints in tumours: inducing blood vessel normalization and the neogenesis of HEV

It is over 40 years since Judah Folkman first proposed that tumour growth could be restricted by blocking the growth of blood vessels that supply the tumour [47]. Since that time intense research activity has identified vascular endothelial growth factor (VEGF) as a primary angiogenic stimulus which is produced by tumour cells in response to hypoxia. There has been a growing realization that hypoxia also fuels the immunosuppressive tumour microenvironment, in part due to accumulation of immunosuppressive leucocytes such as Foxp3^+^ regulatory T-cells (Tregs) [48]. The use of different anti-angiogenic therapies has revealed that, instead of preventing blood vessel growth, sub-optimal doses ‘normalize’ or mature tumour blood vessels by promoting pericyte recruitment and increasing vessel perfusion which may improve oxygenation and remove suppressive metabolites from the tumour microenvironment [49]. Vascular normalization is associated with a reversal of EC anergy, increased effector T-cell infiltration and control of tumour growth in both autochthonous and transplanted cancers, establishing a robust link between vascular normalization and improved T-cell-dependent anti-cancer immunity [50,51]. Tregs suppress blood vessel normalization [52] but a range of inflammatory stimuli including tumour EC-targeted TNF-α [53,54], eosinophils [55], irradiation [56] and TLR ligands [57], in the presence or absence of transferred tumour specific T-cells, can overcome Treg suppression and induce vessel normalization and homing molecule expression on tumour EC. Hyperthermia induced IL-6 trans-signalling and blockade of endothelin B receptor in tumour EC also up-regulate ICAM-1 expression and promote CTL recruitment [22,32]. There is a growing realization that chemotherapy induces immune-mediated destruction of cancer cells but whether tumour blood vessel normalization is involved remains to be determined.

An exquisite example of tumour blood vessel maturation is the development (neogensis) of specialized HEV blood vessels. Studies in mice suggest that, similar to tumour blood vessel normalization, HEV neogenesis is regulated by type 1 inflammation [25] and can be restrained by Tregs [58]. In marked contrast with HEV development in lymph nodes which depends on dendritic cell expressed LTαβ engagement of LTβR in EC, HEV neogenesis in mouse tumours is driven by CD8^+^ T- and/or NK cell secretion of LTα3 and IFN-γ which induce expression of peripheral node addressin (PNAd, a ligand for L-selectin) and the arrest chemokine CCL21 respectively. Although comprising <10% of the tumour vascular network, newly formed HEV in mice recruit adoptively transferred naïve T-cells directly from the bloodstream into the tumour where cross-presenting APC activate naïve T-cells to kill tumour tissue [25]. The development of HEV establishes a site of T-cell priming and re-activation inside cancerous tissues and avoids the loss of tumouricidal T-cells to non-cancerous tissues during redistribution from the normal site of priming in lymph nodes [59]. Interestingly, expression of the HEV-specific marker, PNAd, is below the level of detection using conventional immunohistochemical staining in some tumours and EC lining HEV expressing low levels of PNAd are flat [25], as opposed to the characteristic, LTβR-dependent cuboidal morphology of HEV in LNs [60] which suggests that they are not fully differentiated. The extent and role of HEV development in clinical cancers warrant further investigation as a potential entry site for central memory CAR and TCR_gm_ T-cells which are predicted to have superior efficacy in adoptive cell therapies.

## Summary, outstanding issues and future directions

Although the promise of CAR T-cell therapy for the treatment of solid cancers has yet to be realized, it is clear that adoptive immunotherapy using T-cells expressing conventional TCRs does not work for all patients. Inefficient recruitment by immature tumour blood vessels and lack of persistence of injected T-cells are important determinants of successful outcomes in experimental mice. Manipulating homing addresses on T-cells and vascular addresses on tumour blood vessels to facilitate the recruitment of tumour-specific T-cells offer a combined approach to overcome these limitations in cancer patients. However, longitudinal imaging of transferred T-cells using non-invasive imaging using PET/SPECT tracers will be required to determine whether homing and persistence of transferred T-cells correlate with therapeutic outcomes.

## Abbreviations

ACT: adoptive cell therapy
CAR: chimaeric antibody receptor
CTL: cytotoxic T-lymphocyte
EC: endothelial cells
HEV: high endothelial venule
ICAM-1: intercellular adhesion molecule-1
LPS: lipopolysaccharide
LT: lymphotoxin
NK: natural killer
PNAd: peripheral node addressin
TCR: T cell receptor
TIL: tumour infiltrating lymphocyte
TNF-α: tumour necrosis factor-α
